# Providing HIV testing in men who have sex with men through a gay social networking app in China: A qualitative interview study with multisectoral service providers

**DOI:** 10.3389/fpubh.2022.1056720

**Published:** 2022-11-17

**Authors:** Tianming Zhao, Zhaobing Cao, Zhen Jiang, Gabriella Bulloch, Yanchao Qiu, Lihua Liu, Lijing Wang, Yingxia Li, Ce Jia, Li Guo, Zunyou Wu

**Affiliations:** ^1^Department of Epidemiology and Health Statistics, School of Public Health, Anhui Medical University, Hefei, China; ^2^National Center for AIDS/STD Control and Prevention, Chinese Center for Disease Control and Prevention, Beijing, China; ^3^Faculty of Medicine, Dentistry and Health Sciences, University of Melbourne, Melbourne, VIC, Australia; ^4^Shijiazhuang Municipal Center for Disease Control and Prevention, Shijiazhuang, China; ^5^Shijiazhuang Fifth Hospital, Shijiazhuang, China; ^6^The “Tongxing” Non-Governmental Organization, Shijiazhuang, China

**Keywords:** AHTS, MSM, facilitators, barriers, qualitative interviews

## Abstract

**Objective:**

We explored the feasibility of app-based HIV testing services (AHTS) among men who have sex with men (MSM) according to the perspectives of testing service providers.

**Methods:**

Twenty-one current or previous AHTS providers participated in a semi-structured interview which explored the facilitators and barriers to AHTS. Ten participants originating from the Center for Disease Control and Prevention (CDC) and 11 from the non-governmental organization (NGO) in Shijiazhuang, China took part in this study. Interviews was transcribed verbatim, and the socioecological model (SEM) was applied to thematic analysis.

**Results:**

Twenty-one participants from AHTS services commented on the integration of online appointment schedules into HIV testing services. AHTS was deemed a convenient and efficient method for MSM and service providers to choose their preferred location and times. Moreover, it allowed for important HIV-related information to be accessed online and targeted toward at-risk individuals. Participants thought MSM may feel unsure about personal information security being stored within a privatized app and was considered a barrier to AHTS's usability. As such, they believed establishing a government-led national online platform for AHTS would allow for greater trust from MSM, rather than a social media app.

**Conclusion:**

By linking booking services to an online platform, AHTS was deemed a convenient and efficient method for HIV testing services, especially for young MSM who are familiar with smartphone applications. To improve the use of these services, AHTS apps should focus on ensuring the confidentiality of personal information and internet security to build trust between MSM and service providers.

## Introduction

In China, HIV (human immunodeficiency virus) has become an epidemic among men who have sex with men (MSM) and poses a serious public health challenge (1, 2). Although China has improved the prevention of HIV through prophylaxis treatments and prevention strategies, its prevalence was estimated to be 6% in 2020 (3, 4). A general lack of awareness about HIV and strategies to prevent transmission indicate better strategies are needed to contain outbreaks and lower prevalence further (3).

HIV testing is key for prevention of HIV infections and progression to AIDS, as it allows HIV positive individuals to be identified and treated such that the infection remains inactive and non-transmissible, and provides the opportunity for health providers to encourage protective sexual practices (3, 5). It is estimated only 62.2% of MSM in China have tested for HIV and know their infection status in 2020 (4), indicating HIV testing rates are suboptimal. Disturbingly, a survey conducted in Ningbo, China showed just 57.1% of MSM underwent HIV testing in the past year (6), and in Guangzhou only 44.3% of MSM were tested frequently (testing at least two times per year) over 2 years (7). Moreover, young MSM have a higher sex drive and are more likely to engage in risky sexual intercourse following online activity (5, 8). Concerningly, a previous study on university students found amongst 375 MSM, just 50.4% had ever taken an HIV test (9). Therefore, HIV testing among young MSM should be promoted by public health campaigns to establish a social norm that healthy testing habit is important to lowering HIV rates and risk in China (10–12).

With the advent of social media, its widespread use has also changed dating and sex dynamics among MSM. Settings where MSM date have shifted from traditional locations (bars, saunas, parks, etc.,) to online interactions hence, gay social media apps have been targeted for the promotion and services of HIV testing as a novel way to increase awareness and social normalcy of testing (13–15). Several studies suggest this approach could improve health outcomes by broadening HIV testing coverage and is considered a promising approach (16–22). For example, a Beijing study has found providing online booking systems for HIV testing at a nearby clinics *via* social media platforms significantly increased testing uptake (22). Despite the promise, perspectives of service providers from the CDC and MSM non-government organizations are unknown. Understanding the perspectives of services providers could fine tune this approach and facilitate its implementation in other areas of China. From February 2019 onwards, app-based HIV testing services (AHTS) has been trialed for use in Shijiazhuang, China. This study aimed to explore the experiences of providers and understand facilitators and barriers for providing AHTS to MSM in China.

## Materials and methods

### The process of app-based HIV testing

In 2019, with support of the National Key Science and Technology fund, the Chinese CDC cooperated with Blued, a Chinese gay dating app used in China (1), to set up a HIV booking platform called “Happy Testing”. Here, MSM could choose AHTS sites nearby (CDCs or NGOs) for HIV services. To make the appointment, individuals signed an informed consent form and structured questionnaire that asked about their demographics and assessed risky behaviors. After submitting the questionnaire, individuals could make an appointment for a testing site location and time. After they received a booking code, which would be used to attend their allocated service.

### Study participants

From December 2021 to January 2022, AHTS provider staff were enrolled through two senior staff members from Shijiazhuang CDC and the head of NGO institutions. Participants included in our study from CDC and NGO were recruited by leaders in the institutions independently based on the following criteria: (1) currently or formerly engaged in AHTS (work on publicity, counseling or testing of AHTS) for MSM and (2) willing to participate in our interview.

### Data collection

Data was collected through the means of individual semi-structured interviews. Each interview was 30–60-min long and done *via* an online meeting or phone call. The goal of the meeting was to explore the experiences and perspectives of delivering AHTS to MSM. Questions were open-ended and examples are as follows: Can you describe the process of providing AHTS services to MSM? Describe what issues you encountered when providing AHTS services? Describe the strengths of this service based on your own experience. After acquiring verbal consent, all interviews were audio-recorded by a recording pen and transcribed verbatim using the transcription application iFLYREC (https://www.iflyrec.com/software). All interviews were conducted by TZ and ZC. Then, audio recordings and transcriptions were reviewed by TZ and ZC to check the accuracy and completeness of data. Data about the participants were translated to a unique ID code which allowed for the institution type and location to be referenced to throughout analysis.

### Data analysis

Qualitative analyses of interviews were conducted by means of a thematic analysis approach which was guided by the socioecological model (SEM) (23–25). Transcriptions, coding, and analyses in this study were completed in Mandarin, and results were translated to English for manuscript preparation (26). Thematic analysis identified themes within the transcription data through a set of standardized steps: (1) the researcher familiarized themselves with the data by reading transcripts multiple times and listening to audiotapes; (2) using deductive and inductive methods, codes were generated according to things that appeared meaningful and identified any possible themes. These themes were reviewed, named, and identified as facilitators or barriers of AHTS service. Specific quotes were also taken from the transcribed interviews as representative examples during analysis. (3) using SEM, themes were categorized into individual, interpersonal, organizational, community, and public policy levels according to how they would influence the future of AHTS (23–26). All analyses were conducted by TZ and ZC through NVivo qualitative data analysis software.

## Results

### General characteristics of participants

A total of 21 AHTS providers were recruited and completed the interview (from NGO and CDC in Shijiazhuang), which consisted of 10 individuals from CDC and 11 individuals from NGO. The average age of all participants was 39.43 years (range, 19.99–70.35 years). Participants from CDCs had an average age of 48.71 years (34.52–70.35 years), and this was 31.69 years in NGOs (19.99–45.14 years). From the analysis of interviews, 18 main themes emerged which was categorized into five dimensions based on SEM analysis (individual, interpersonal, organizational, community and public policy, Table 1).

**Table 1:** Multilevel influencing factors of providing AHTS in MSM.

**Individual**	**Interpersonal**	**Organizational**	**Community**	**Public policy**
**Facilitators**				
Acceptability of online information system	Facilitating peer mobilization	Digital management	The dissemination of AHTS-related information	Stimulating the evaluation of HIV testing policy
Convenience in services		Enhancing testing efficiency		
		Covering high-risk MSM		
**Barriers**				
Operations of online services	Difficulties in effective communication	Heavy workload	Distribution of AHTS sites	The authority of online platform
The usage of online platform			Effect of other testing services	Effect of COVID-19 related policies on AHTS
Personal characteristics of MSM				
Trust of testing facilities				

### Individual facilitators of providing AHTS

Participants in our study mentioned filling out online information for HIV risk assessment was more consumer friendly than face-to-face questioning, as MSM would feel less embarrassed and reserved about their answers. It was agreed that online HIV risk assessment made MSM feel less judged by their responses and allowed them to answer questions at their own pace. This also improved information accuracy most of the time. “*When filling out information online, they (MSM) may not feel pressured about their response because no one else is present and there can be no judgement. This makes the user more relaxed about completing the questionnaire.” “If they feel insecure about their response to a question, there is no one online to judge their answers- they likely don't feel worried about giving this information.”* Furthermore, AHTS services was convenient for MSM, with uploads of past testing appointments on the app allowing users to determine whether they need testing and when they last had a test. Additionally, AHTS services also allowed them to select suitable times and see nearby locations in the one app, rather than having to check nearby locations individually online and calling to find their individual clinic availabilities. This was also deemed convenient and flexible. “*If they (MSM) forgot the last time they were tested, they can log in and see the date of their last appointment. This information can confirm whether they need to be tested again.” “Some (MSM) may have work, school or something like that- they can arrange the testing time according to their own situation”*.

Barriers for individuals using AHTS included the operation of platform login and information filling, which may hinder use by older MSM, those who were vision impaired, or people unfamiliar with using online services. Several members expressed concerns about whether some MSM could complete the online booking process and questionnaires needed for AHTS and expressed this may impact the accuracy of online information collection if the user is unsupervised or not helped. “*Some middle-aged and elderly people use the social media app and know about AHTS through the platform, but they don't understand its functions and online questionnaires. Some people have told me they (MSM) want to make an appointment online, but don't know how to do it.” “Some elderly MSM don't know how to use smartphones, or can only use cellphones which cannot make an online appointment.” “MSM may finish the online questions, but the reliability of content is uncertain. After all, it is not face-to-face, we cannot tell whether or not he was able to understand the question and answer correctly.” “There is also a problem with the authenticity of the information filled online. They won't necessarily give you real information.”* Some staff stated the awareness of the social media app among MSM was limited and affected the accessibility of AHTS. “*If MSM use the social media app, they can make an appointment but for MSM who don't know or don't use it, they lose access.” “Some people also may have used the social media app for a while but might delete it.”* Personal characteristics of MSM including psychological factors and personal mobility also impacted the uptake of AHTS. Online services also do not change people's mindset toward HIV testing and fear of a positive result which are at the crux of the testing issues. “*I think that some MSM are in denial. They feel they will be fine and do not need testing.” “Some MSM are just afraid of being diagnosed with HIV, and to avoid problems and reality, they do not test.”* In addition, personal information privacy was a concern. “*Some MSM may think information on the Internet is not secure and are afraid of privacy leaks.” “Some MSM may worry about whether their information may be leaked and whether personal privacy is respected online.”* Mobility of MSM also affects the continual uptake of AHTS services due to geographic breadth of the service which could complicate reliable testing data “*There may be relatively large individual turnover. He is staying here now, and he will come here to test, and when he goes to other places next year, he will go to other places to test.”* MSM's trust in testing facilities also exerted a significant influence on AHTS seeking. Some participants expressed the varied trust in CDCs and NGOs among MSM. “*I feel that this is an inherent thought that health institutions affiliated by provincial governments (higher institutional level) were better than their counterparts affiliated by municipal governments (lower institutional level).” “For district-level CDC which are close to municipal CDC, most of MSM are less likely to go to the district CDC.” “Many MSM tended to choose their familiar NGOs.” “Some MSM remain skeptical of the authority of the NGO”*.

### Interpersonal-level facilitators and barriers of providing AHTS

Several participants mentioned MSM who used AHTS services could facilitate other MSM through word of mouth, which may promote the uptake and awareness of HIV testing through AHTS. “*Information spreads quickly online, and many people receiving AHTS may introduce it to their friends.” “Some MSM may introduce the app to their friends if they have a positive experience with AHTS services. It is very convenient”*.

Besides, several members from CDCs reported non–MSM service providers found interpersonal communication difficult. “*It is not easy to intervene for certain groups (of MSM). I think it is difficult for a heterosexual worker to intervene in this group of people. It is not easy to be trusted by his small circle”*.

### Organizational-level facilitators and barriers of providing AHTS

Staff included in our study also commented that online booking made HIV services more sustainable, standardized, and accessible. “*Data collection from online platforms are very good because we can see how many tests are done per year and can better understand individual testing and risk status.” “I think in the information age, the storage of information online is more convenient than by paper.”* Online scheduled services also help HIV testing staff individuals and service agencies optimize their schedules and provision of services, which improves the service efficiency for testing facilities. “*For ourselves, we can arrange work schedules in advance according to appointment times.” “I can arrange my own time reasonably according to the service schedule of the testing point.”* Some participants in our study indicated users of social media apps tend to have more high-risk sexual behaviors including multiple sexual partners and low condom use. This implies promotion of AHTS is sensitive to vulnerable populations with better public health impacts. “*Many young people use social media apps, and their sexual activity and desire to make friends is also high. They are also more likely to have casual sexual partners. If they are more sexually active, the risk of their sexual behaviors may also be high”*.

Many participants reported organizational challenges for providing AHTS. Specifically, staff from the CDCs commented that AHTS increased the demand for testing and counseling. The added workload was not met with adequate staff, which caused service shortfalls. During the COVID-19 pandemic, many CDC staff participated in epidemic prevention and control, which restricted time and effort devoted to AHTS. Moreover, the fixed working time restricted the number of AHTS services provided by CDCs. “*During the COVID-19 pandemic, most of our energy was redirected. I was responsible for other tasks. With HIV test bookings online, sometimes there was no time or energy for the service. Some people came for their appointed time and staff wasn't there- this causes trouble.” “The service quality of AHTS is restricted by number of personnel. We may have other jobs after an online appointment was made. If that happens, we cannot provide services for them.” “If someone wanted to go on a weekend or at night, NGOs or hospital were the only other option”*.

### Community-level facilitators and barriers of providing AHTS

Some participants commented online platforms could facilitate the dissemination of AHTS-related information in the social network of MSM, which could help cultivate the online HIV testing culture in local MSM community. “*I think the online platform has also promoted HIV testing. To be specific, more people know about AHTS. Because not all people could see our offline campaigns. Through the Internet, more people know about it.” “AHTS helps cover a wider range of MSM, and the volume of HIV testing will increase”*.

However, the unbalanced regional distribution of testing sites in communities is an important factor affecting the accessibility of services. Since the AHTS sites were mainly located in urban areas, this limited the availability of services in rural areas. “*Our AHTS sites are mainly in urban areas, but MSM are also in rural areas, and it is not convenient for them to seek AHTS through NGO or CDC.”* The staff also stated the existence of other service resources makes AHTS less needed in city centers. “*We have many testing sites here, and the HIV testing can be done in many other places. It is not necessary for him to seek for AHTS.” “If they don't want to seek AHTS, they could also get HIV self-test kits”*.

### Policy-level facilitators and barriers of providing AHTS

Participants conveyed AHTS's online nature provides the potential to improve HIV testing services, and shared opinions on how to best optimize AHTS as a public health strategy in the future. In particular, enhancing accessibility to rural areas and strengthening NGO service quality was a priority. “*AHTS has shown potential to promote HIV testing in MSM and expanding the geographical coverage of AHTS sites is being considered.” “Enhancing the supervision and guidance for NGO is needed to improve their service quality”*.

Providers also suggested several times the construction of a nationally led online HIV service information and booking platform would improve the reliability and trustworthiness of AHTS. Providers thought if the platform was supported by the government, it could facilitate the enthusiasm of health facilities in other geographical regions to join in and increase the legitimacy of the service. “*I reckon it is better to choose a platform which is more authoritative. If the platform was built by the government, I believe that the MSM will feel it more trustworthy, and CDCs in various areas will be willing to join this platform.”* Furthermore, COVID-19 lockdown policies reduced the availability of HIV testing, which reduced the feasibility of MSM to consider AHTS. “*Due to COVID-19, the CDC has restricted access to testing, and they (MSM) cannot be tested.” “For some college students, school is closed, and they cannot come out because of COVID-19.” “Because of the impact of the COVID-19, students cannot leave school. Even if they want to test, they just can't get out”*.

## Discussion

This study provides detailed accounts about the facilitators and barriers to AHTS and their impact on HIV testing in China. The app was considered a consumer-friendly and convenient method for HIV testing, however user information security and concerns about legitimacy require improving for improving AHTS uptake. The suggested changes proposed by service providers are important to the future of HIV booking services and may be important information for other countries and software engineers to consider when creating a user-friendly platform.

Across service providers, participants in this study believed AHTS improved positive testing and normalized HIV testing culture in MSM communities by increasing its exposure on a gay dating app and making testing services publicly available. Providers thought the nature of AHTS allowed for a people-centered approach as the individual had the power of choosing which HIV testing service or time worked for them. In addition, self-risk assessment and remote questionnaires allowed the individual to feel control over the HIV testing process. This was a positive take-away point to note, as person-centered frameworks have been touted by the World Health Organization as facilitators to care, improve health and clinical outcomes, health literacy and satisfaction (27, 28). Qualitative studies also suggest the use of online booking services creates a positive individual user-experience because the person is not required to hold or wait while on a call, answer questions verbally in public, and allows for in-advance scheduling which may not be available otherwise (29).

Despite this, health providers reported the quality of online information, especially risky behaviors, could be impacted if active supervision wasn't provided. This is worrying, considering the reliability and validity of information in HIV risk assessment is important to ensuring risk-stratification tools are properly incentivized and tailored for the individual, for example the recommendation of pre-exposure prophylaxis or increased frequency of testing (30). Although self-administrated risk assessments were expressed by some providers and previous qualitative studies (29) to reduce judgement and provide more convenient risk assessment of HIV infection, self-reported information is subject to recall and social desirability biases, which may not reflect one's reality (31, 32). This could be made worse without effective face-to-face communication, which reduces the service quality and authenticity of information. Although this was a concern by service providers, a qualitative analysis of HIV testing app users in South Africa and Canada expressed app-based risk assessment resulted in less fear of answering questions, which was attributed to the less personable nature of answering a phone-based questionnaire (29). In addition, fear of judgement from in-person assessors led some users to forgo testing because they weren't mentally ready for face-to-face assessment. To reduce this problem, information re-checking on a printed version of the user's responses once arriving at the clinic would assist the quality of data, but some degree of self-reporting bias will always exist whether face-to-face or online questionnaires are used.

While on one hand AHTS allows for convenient and people-centered approaches to HIV testing, the stigma and discrimination associated with HIV testing is a barrier that prevents some MSM from seeking care. Being on a social media app, the knowledge and awareness about AHTS and HIV testing is disseminated easier and faster, which for some, could exacerbate insecurities about a possible or diagnosed positive status. Furthermore, worries about personal information security have been associated with fears of discrimination and stigma, and likely influence negative views on AHTS services (33). Therefore, while AHTS is a vehicle to improve testing, the underlying social stigma, confidentiality, and privacy concerns require specific addressing from a public health perspective (34–36). Health promotion advocacy should seek to decrease fears associated with HIV and increase the effectiveness of HIV treatments which strop progression and reduce transmission (37). Additionally, training programs which develop non-judgmental and empathetic communication skills for providers will help eliminate feelings of estrangement and build trust between MSM and testing facilities.

The theme of barriers to access was prevalent across interviews, and several members expressed a lack of AHTS sites in rural areas limited the volume of AHTS uptake. Considering geographic distance is associated with HIV testing services rates (38), expanding AHTS to rural areas would facilitate utilization of HIV testing in the future. Access to AHTS for MSM without access to the app, who were elderly, or traveled regularly (for work, truck drivers, etc.,) was an additional concern as AHTS was only available locally (39, 40). Clearly, while AHTS has the potential to increase HIV testing among MSM, this strategy has inherent restrictions which currently cannot replace other means of booking. Considering AHTS also limited services to HIV testing, the addition of other HIV-related appointments like voluntary counseling and testing, provider-initiated testing and counseling and HIV self-testing was suggested to allow MSM access to other services from one provider. This would allow for continuity of care and a sense of support from their provider.

Information security was a prevalent theme that provider representatives thought would limit the uptake of AHTS. Considering AHTS services are only available through a social media app, worries about the perceived authority of the app and guaranteed confidentiality are reasonable. These concerns are not unique to AHTS providers, with a recent BMJ report finding serious problems with privacy in mobile health apps where lack of information safety could permit third parties from accessing user emails, and 88% could access and potentially share personal data (41). In view of this, AHTS providers suggested a government-led platform would assure both MSM and providers about the authenticity of HIV testing considering the government is a highly regarded institution amongst Chinese citizens (42, 43). This would also likely allow for better security and privacy technology to be integrated within the platform, thereby relieving fears about information leaks or third-party usership (41).

During the COVID-19 pandemic, some CDC staff responsible for HIV prevention were transferred to different roles to control the spread of severe acute respiratory syndrome coronavirus 2 (SARS-CoV-2). This impacted the capacity of CDC for providing AHTS, while the COVID-19 lockdown policy also restricted MSM from accessing HIV testing services. This effected the accessibility to HIV testing services and communication of availability within the app. In future, providers and facilities should work together to further improve communication and streamline the experience for HIV testing services (44, 45). The addition of HIV self-testing to AHTS is an alternative which could improve the accessibility of HIV testing services and meet the testing needs of MSM during lockdowns (46). Testing agencies should consider recruiting more professionals to improve their management and ensure the continuity of HIV testing services.

This study provides useful feedback which indicates integrating online services into HIV testing strategies could expand HIV testing by providing convenience to a service that is otherwise stressful and difficult to coordinate in China. While having AHTS present on a gay dating app does promote awareness about HIV testing, the information gained by this thematic analysis suggests a government-led platform would improve perceived trustworthiness and legitimacy about AHTS. It would therefore be in the best interests of public health to facilitate this coordination, and meanwhile also consider expanding services to neighboring rural areas where HIV testing services are not as plentiful. It would be useful if future interventions investigated the perceived legitimacy of a government-led platform for information security and public credibility to investigate whether these interventions were successful, should they be implemented.

## Strengths and limitations

Some limitations of this study should be acknowledged. First, this study focused on AHTS services among providers in Shijiazhuang which limits the generalizability of these findings to wider China and other continents. Second, the small sample size in our study (*n* = 21) prevents this thematic assessment from providing a robust initial assessment. Despite this, the heterogeneity of themes presented by the participants suggests this study achieved thematic saturation, hence no further participants were sought after interviews were completed. Thirdly, interviews were conducted on past service providers and not MSM users of AHTS. This likely influences the depth of data analyzed in this study and could limit the validity of statements pertaining to MSM experiences. As such, providers can only comment on their own opinions or what MSM have stated in the past, which may not be representative of MSM. It would be beneficial to have both opinions available for comparison, and these may allow for changes to AHTS which could be tailored to MSM.

## Conclusion

Although AHTS has several advantages, a series of challenges remains to be addressed. Future interventions should emphasize confidentiality and establish a government-led approach to AHTS to address MSM concerns. Future studies should interview MSM to gauge their own experiences and concerns to provide a more tailored approach to AHTS that facilitates greater support and uptake of HIV testing.

## Data availability statement

The datasets presented in this article are not readily available because the datasets generated and/or analyzed during the current study are not publicly available due to privacy and confidentiality agreements as well as other restrictions but are available from the corresponding author (ZW) on reasonable request. Requests to access the datasets should be directed to ZW, wuzy@263.net.

## Ethics statement

This study was approved by Institutional Review Board of National Center for AIDS/STD Control and Prevention, China CDC (NCAIDS/STD, Project No.: X180629516).

## Author contributions

TZ, ZJ, YQ, LL, LW, YL, CJ, and LG contributed to the conception and design of the study. TZ and ZC contributed to the collection and analysis of data. TZ wrote the first draft of the manuscript. ZW and GB critically reviewed and revised the manuscript. All authors read and approved the final manuscript.

## Funding

This work was supported by National Science and Technology Major Project of China (2018ZX10721102) and Research on the model of intensive HIV prevention and control strategy among men who have sex with men supported by National Natural Science Fund–Integrated cost-effectiveness analysis based on the natural cohort (71874169).

## Conflict of interest

The authors declare that the research was conducted in the absence of any commercial or financial relationships that could be construed as a potential conflict of interest.

## Publisher's note

All claims expressed in this article are solely those of the authors and do not necessarily represent those of their affiliated organizations, or those of the publisher, the editors and the reviewers. Any product that may be evaluated in this article, or claim that may be made by its manufacturer, is not guaranteed or endorsed by the publisher.
